# Viral Interactions with Adaptor-Protein Complexes: A Ubiquitous Trait among Viral Species

**DOI:** 10.3390/ijms22105274

**Published:** 2021-05-17

**Authors:** Ivana Strazic Geljic, Paola Kucan Brlic, Lucija Musak, Dubravka Karner, Andreja Ambriović-Ristov, Stipan Jonjic, Peter Schu, Tihana Lenac Rovis

**Affiliations:** 1 Center for Proteomics, Faculty of Medicine, University of Rijeka, 51000 Rijeka, Croatia; paola.kucan@medri.uniri.hr (P.K.B.); dubravka.karner@medri.uniri.hr (D.K.); stipan.jonjic@medri.uniri.hr (S.J.); 2 Fidelta Ltd., Prilaz baruna Filipovica 29, 10000 Zagreb, Croatia; Ivana.StrazicGeljic@fidelta.eu (I.S.G.); Lucija.Musak@fidelta.eu (L.M.); 3 Department of Histology and Embryology, Faculty of Medicine, University of Rijeka, 51000 Rijeka, Croatia; 4 Laboratory for Cell Biology and Signalling, Division of Molecular Biology, Ruđer Bošković Institute, Bijenička 54, 10000 Zagreb, Croatia; Andreja.Ambriovic.Ristov@irb.hr; 5 Department of Cellular Biochemistry, University Medical Center, Georg-August-University Göttingen, Humboldtallee 23, D-37073 Göttingen, Germany; pschu@gwdg.de

**Keywords:** adaptor protein complexes, protein sorting, endocytosis, herpesviruses, HIV, respiratory viruses, hemorrhagic fever viruses

## Abstract

Numerous viruses hijack cellular protein trafficking pathways to mediate cell entry or to rearrange membrane structures thereby promoting viral replication and antagonizing the immune response. Adaptor protein complexes (AP), which mediate protein sorting in endocytic and secretory transport pathways, are one of the conserved viral targets with many viruses possessing AP-interacting motifs. We present here different mechanisms of viral interference with AP complexes and the functional consequences that allow for efficient viral propagation and evasion of host immune defense. The ubiquity of this phenomenon is evidenced by the fact that there are representatives for AP interference in all major viral families, covered in this review. The best described examples are interactions of human immunodeficiency virus and human herpesviruses with AP complexes. Several other viruses, like Ebola, Nipah, and SARS-CoV-2, are pointed out as high priority disease-causative agents supporting the need for deeper understanding of virus-AP interplay which can be exploited in the design of novel antiviral therapies.

## 1. Introduction

Viruses have evolved a plethora of strategies to counteract the mechanisms of host’s immune response at various stages of their life cycles. From internalization of viral particles and replication of the viral genome to viral assembly and release of mature virions, the interference with cellular trafficking and sorting machinery appears to be an asset for viral propagation and antagonizing of host antiviral response [1]. Clathrin-mediated endocytosis (CME), a major cellular ubiquitous route of receptor internalization, is a common route utilized by viruses, particularly of small and intermediate size. Regardless of the viral receptor used, CME is dependent on the oligomeric clathrin and adaptor-protein (AP) complexes that coordinate the recruitment of endocytic cargo proteins and assembly of clathrin into a polyhedral lattice at the plasma membrane and the trans-Golgi network (TGN) [2,3,4,5,6]. A large body of evidence showed that many viral genera take advantage of cellular AP complexes, making them conserved viral targets [7].

We will present different mechanisms of viral interference with cellular AP complexes and their functional consequences that allow for efficient viral propagation and evasion of host’s immune defense. Given the fact that the International Committee on Taxonomy of Viruses currently recognizes over 5500 viral species [8], this review will focus on viruses known to cause infections in humans and the most commonly studied animal viruses. Viruses discussed in the review are listed in Table 1 and presented following the Baltimore classification of viruses which is based on the structure of the viral genome and the manner of messenger RNA (mRNA) synthesis.

## 2. Main Body

### 2.1. Composition and Function of AP Complexes

**Figure 1:**
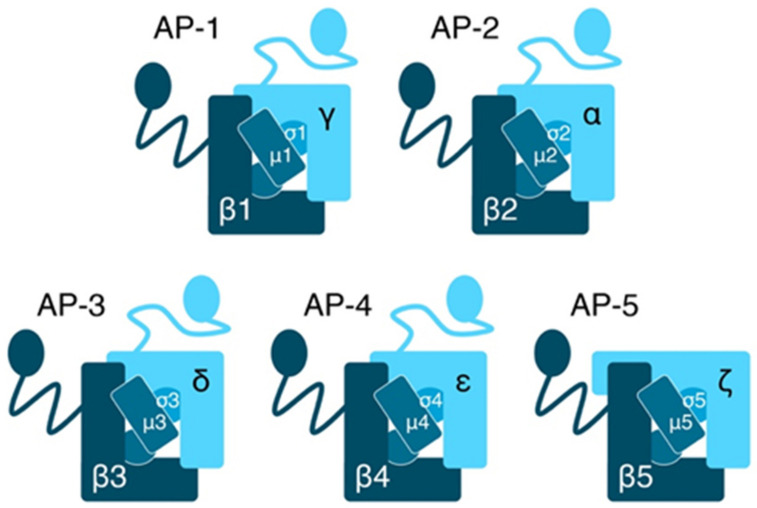
Family of AP-complexes and their subunit composition. Each complex is composed of two large adaptins, one medium-sized μ-adaptin and one small σ-adaptin.

APs are a family of heterotetrameric protein complexes that mediate the sorting of proteins by vesicles in endocytic and post-Golgi secretory transport pathways [2]. To date, five different AP complexes have been described [4,5] with specific localization to intracellular compartments and distinct functions in protein sorting and transport (Figure 1). AP-1 and AP-2 mediate the formation of clathrin-coated transport vesicles, while AP-3, AP-4 and AP-5 mediate protein transport independent of clathrin [2,3,4,9]. The numbering reflects the chronological order of their discovery. Structurally, all AP complexes are composed of two large adaptins, one medium-sized μ-adaptin and one small σ-adaptin (Figure 1). Both large adaptins consist of a *N*-terminal core domain, followed by a long unstructured hinge domain and a small C-terminal globular domain, also referred to as ear domains [10,11]. The large β-adaptins of the AP-complexes, β1 to β5, have the highest sequence homologies of the AP-adaptins. Their ear domains bind and recruit additional coat proteins, which bind cargo proteins not recognized by the AP-complex. The β1 and β2 ear domains bind clathrin [9,12,13]. The second large AP adaptins show the least sequence homologies and were named γ, α, δ, ε, and ζ (AP-1 to -5). Their core domains mediate AP organelle specific membrane binding and their ear domains mediate binding to so called accessory-proteins and coadaptor proteins. Most cargo proteins carry canonical sorting sequence motifs in cytoplasmic domains recognized by the AP complex μ- and σ-adaptins.

Isoforms exist of some adaptins of AP-1, AP-2, and AP-3 complexes. AP-2 has two ubiquitous α isoforms (αA and αC) and AP-3 has one ubiquitous and one tissue specific isoform of β3 (β3A and β3B), μ3 (μ3A and μ3B) and σ3 (σ3A and σ3B) [2,4]. AP-1 complexes form the largest AP family. γ1AP-1 and γ2AP-1 complexes are ubiquitously expressed, and both contain β1, μ1A and σ1A adaptins [14]. These complexes fulfil indispensable, nonoverlapping functions in early vertebrate development [14,15]. In γ1AP-1, σ1A subunit can be replaced by the tissue-specific σ1B or σ1C, whereas σ1A of γ2AP-1 can be replaced only by σ1B [16,17]. Some viruses use tissue-specific adaptin expression. For example, polarized cells of epithelia express an additional AP-1/μ1B isoform [18], whose absence confers higher susceptibility to viral infection, as further elaborated for HAdV. 

AP complexes bind numerous cargo proteins based on short sorting motifs present in the cytoplasmic domains of transmembrane proteins. Two canonical sorting motifs have been described: a tyrosine-based motif bound by μ-adaptins and di-leucine-based motifs bound by σ-adaptins. The tyrosine-based sequence is YxxØ (x any amino acid; Ø bulky hydrophobic amino acid), and the di-leucine-based sequences are D/ExxxLL/I. Amino acid residues preceding di-leucine-based motifs determine σ-adaptin binding specificity [19,20,21]. In addition to these, AP complexes bind numerous additional cargo protein sorting motifs, which appear to be less conserved during evolution and we refer here only to some of the described motifs [19,21,22,23,24].

Cargo protein binding stabilizes AP-complex membrane binding, but cargo binding requires a large conformational change of the AP-complexes. The cytoplasmic complexes are in a closed conformation, in which μ- and σ-adaptins are not able to bind sorting sequence motifs due to the steric hindrance. The μ-adaptin C-terminal domain, which contains the YxxØ motif-binding domain, is embedded between large adaptins while phosphorylation of μ1 and μ2, by AAK-1 and/or GAK kinase, enables conformational change and cargo protein binding [25,26,27,28]. The μ-adaptin conformational change enables conformational changes in both large adaptins, which releases the steric blockade of the σ-adaptin di-leucine motif-binding domain [29]. Membrane binding and the open conformation of γ1AP-1 is further supported by its binding to the Arf1-GTP [30]. AP-3 and AP-4 membrane binding is also supported by Arf1-GTP, whereas γ2AP-1 and AP-5 membrane binding is not [3,4]. The role of AP-complex phosphorylation was documented for HCV and EBOV life cycle. In addition, HIV protein Nef is able to reshape, reorganize, and ultimately stabilize AP complex with the target molecule. Simpler viral strategies include inhibition of adaptin and target binding.

The γ1AP-1 forms clathrin-coated vesicles at the TGN and mediates bidirectional TGN-early endosome protein sorting. Major cargo proteins in this pathway are the mannose-6-phosphate receptors (MPR), which bind and sort soluble lysosomal enzymes. The AP-1/μ1A regulates MPR endosome-basolateral plasma membrane recycling and thus the MPR300 endocytic capacity. γ1AP-1/μ1B localizes preferentially to recycling endosomes, where it mediates the basolateral recycling of proteins [31]. The γ1AP-1/μ1B also binds, like γ1AP-1/μ1A, to the TGN and it even can substitute γ1AP-1/μ1A in MPR sorting [32]. Due to these ubiquitous house-keeping functions of MPR300-mediated protein sorting, it is not surprising that several viruses, like HIV-1, VZV, and rotaviruses, bind MPR300 for cell entry via CME, as well as for cell egress [33,34,35,36,37]. Another excellent example of exploiting the specificity of AP-1 are HCV and herpesviruses that skillfully select adaptins to avoid apical surfaces and avoid host immune response.

AP-2 is indispensable for early vertebrate development, like the AP-1 complexes, and it mediates exclusively the endocytosis of plasma membrane proteins via clathrin-coated vesicles. After their clathrin coat has been disassembled, these vesicles fuse with early endosomes. AP-2 binds numerous coadaptor proteins, which bind cargo proteins not recognized by AP-2 adaptins. Therefore, AP-2 clathrin-coated vesicles have the largest cargo repertoire of all AP-complex transport vesicles [19,38,39]. Recently, it has been demonstrated that the AP-2 coadaptor proteins, Eps15L1, and epsin-1 form specialized endocytic clathrin-coated vesicles independent of AP-2 [40]. Given the central function of AP-2 in plasma membrane protein endocytosis, it is not surprising that cell entry of multiple viruses depends on AP-2, like human rhinoviruses [41] or in combination with AP-2 coadaptors, such as Eps15 for VACV [42] as well as DAB2 in the case of EBOV [43].

The AP-3 complex protein sorting function is essential for the biogenesis of secretory, lysosome-like organelles, like melanosomes and dense-core vesicles in platelets. In addition, AP-3 supports endosome to lysosome protein transport. In AP-3-deficient cells the lysosomal membrane protein LAMP1 recycles repeatedly between endosomes and the plasma membrane before being delivered to lysosomes [44,45]. The fact that AP-3 directs proteins toward potential lysosomal degradation has been exploited by several viruses to remove host molecules hazardous to their survival. Examples of herpesviruses and MHC I molecules are thoroughly studied [46,47,48]. AP-3 has also been reported to secrete VSV-G, to control HIV-1 Gag trafficking and virus assembly [49,50].

AP-4 localizes preferentially to TGN and endosomal membranes and recognizes YX[FYL][FL]E motifs to sort proteins to basolateral endosomes, like the already mentioned MPRs [51,52]. In neurons, it mediates somatodendritic (basolateral) protein sorting of AMPA receptors [53]. AP-4 sorting of ATG9A contributes to autophagosome maturation [54,55]. Regarding the role of AP-4 in viral life cycle, it was recently documented that it contributes to the release and spread of HCV, along with the AP-1 complex [56]. In addition, the BMRF-2 protein of Epstein-Barr virus is found to exploit μ4 adaptin for its intracellular transport [57].

AP-5 is the latest addition to the AP-complex family. Its deficiency causes spastic paraplegia, due to the degeneration of motor neuron axons and axonal Charcot-Marie-Tooth disease [3,58]. AP-5 forms a complex with two additional spastic paraplegia proteins, SPG11 and SPG15. The complex AP-5/SPG11/SPG15 is localized to late endosomes and lysosomes and is considered to be important in retrieval of Golgi proteins from late endosomes [5,59] and in autophagic lysosome reformation [59,60]. Our knowledge about molecular mechanisms regulating AP-5 functions is still very limited. The expression levels of AP-5 and of AP-4 are very low compared to the levels of the other three AP-complex family members, which makes their analysis more challenging. So far, it was reported only for the HIV 2 that is being transported by AP-3 and AP-5 [61].

### 2.2. Viral Interaction with AP Complexes

#### 2.2.1. Adenoviruses

Human adenoviruses belong to the Mastadenovirus genus of the Adenoviridae family. Up to date there are 67 distinct, naturally occurring human adenovirus serotypes, classified into 7 subgroups (A–G) based on neutralisation with specific antisera or more recently by DNA sequence [8]. In general, adenoviruses cause mild disease and adenovirus-derived replication-deficient vectors have proven promising for tumor gene therapy and vaccination [107,108]. Most human adenoviruses (HAdVs) bind the receptor CAR while some of them bind CD46 [109]. CAR-binding HAdVs use cell surface integrins as secondary receptors which trigger AP-2 CME [110]. In most epithelial cells the AP-1 complexes (AP-1/μ1A and AP-1/μ1B) cooperate in basolateral sorting of CAR [62]. The apical localization of CAR, due to the absence of the AP-1/μ1B in retinal pigment epithelium, allows high susceptibility of these cells to HAdV infection [111].

HAdV transmembrane proteins RIDα and RIDβ form a RID complex [112] which downregulates (i) the EGFR [113,114] and (ii) apoptosis receptors-Fas [115,116], TRAIL-R1 and TRAIL-R2 from the cell surface [117], by rerouting them to endosomes/lysosomes for degradation, thus protecting the infected cells from apoptosis. Both functions are enabled by the interaction of the viral RID complex with AP proteins, where it is interesting to note that the components of the RID complex can bind two different AP proteins and synergize in immune evasion that is beneficial for the virus [63,118]. The mutation of either, the Y122 in RIDβ or the LL87 in RIDα, abolished binding to the AP-1 and AP-2 complexes. RIDβ is necessary for rapid endocytosis of the RID complex, while the RIDα reroutes RID complex into a recycling pathway and protects RID complex from lysosomal degradation [118]. The RIDα also possess the putative tyrosine motif at Y72 whose substitution reduces binding to AP-1 and AP-2, retains RIDα in the TGN and blocks its ability to facilitate EGFR degradation [64]. 

In conclusion, AP proteins are important for HAdV CME, for the basolateral localisation of HAdV receptor CAR and are involved in the modulation of host immune responses by HAdV RID complex.

#### 2.2.2. Herpesviruses

The Herpesviridae family is a large family of double-stranded DNA viruses that are characterized by relatively large genomes with many genes devoted to immune evasion and establishment of latency, which is considered a hallmark of herpesviruses. The family includes at least eight species (HHV-1-HHV-8) that can infect humans and are classified into three subfamilies: α herpesvirinae subfamily includes herpes simplex virus type 1 (HSV-1; HHV-1), HSV-2 (HHV-2) and Varicella Zoster Virus (VZV; HHV-3); β herpesvirinae subfamily includes human betaherpesvirus 6A and 6B (HHV-6A/6B), human betaherpesvirus 7 (HHV7) and human cytomegalovirus (HCMV; HHV-5); and γ herpesvirinae subfamily includes Epstein-Barr virus (EBV; HHV-4) and Kaposi Sarcoma-associated Herpesvirus (KSHV; HHV-8). 

##### Alphaherpesviruses

**Figure 2:**
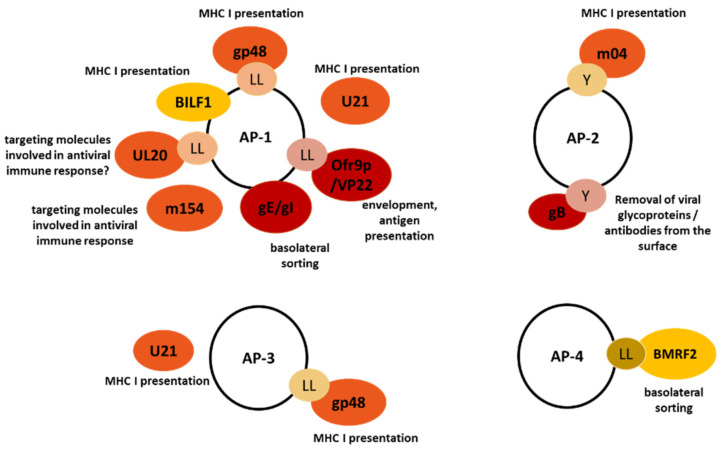
Herpesvirus proteins that have been found to interact with or interfere with AP complexes are presented. α-herpesvirus proteins are shown in red, β-herpesvirus in orange, and γ-herpesvirus in yellow. The function affected by herpesvirus proteins and the motif, if known, are listed.

Strategies associated with AP complexes described for α herpesviruses as mechanisms to avoid recognition by the immune system are (i) direct cell-to-cell spread facilitated by directed basolateral sorting and (ii) controlled surface expression of viral proteins resulting in different immune evasion mechanisms (Figure 2).

The first strategy is used by a conserved heterodimer of glycoproteins gE and gI [119,120]. The gE contains tyrosine and dileucine motifs that interact with AP-1, which allows the accumulation in TGN promoting envelopment of nucleocapsids and budding of virions sorted to lateral surfaces. Authors speculated that AP-1/μ1B -directed movement of viral particles to lateral surfaces serves to increase viral spread to neighboring cells and to avoid contact with the components of the immune system that could be present on the apical (epithelial) surfaces [66]. 

Another glycoprotein, gB, physically interacts with AP-2 via YQRL motif responsible for its spontaneous and antibody-dependent internalization [68]. The internalization of viral surface-associated glycoproteins is conserved among several α herpesviruses and has been proposed as a strategy to avoid immune recognition [121,122,123,124]. Another possibility, as shown for HSV-1 and VZV gE/gI complex, that presents Fc receptor activity, is that antibody-crosslinking of a receptor induces the binding of AP proteins, targeting the bound antibody to the lysosomes for degradation, thus avoiding classical complement pathway [125,126] or antibody-dependent cellular cytotoxicity. For example, in infected monocytes, cells that are essential for viral dissemination, viral envelope proteins are expressed on the plasma membrane but upon antibody binding, they aggregate and are internalized, protecting them from efficient antibody-dependent lysis [127]. The addition of antibodies against gB or gD induced cointernalization of other glycoproteins, including MHC I, which could lead to an impaired cytotoxic T-cell response, suggesting broader mechanisms of immune evasion [128].

Finally, although the interaction has been mostly described for viral envelope glycoproteins, recent publication showed the interaction between VZV tegument protein ORF9p and AP-1, important for the formation of infectious viral particles and viral egress [69]. Even though an immunosubversive role of this interaction has not been yet described for VZV, ORF9p HSV-1 homologue VP22, that also interacts with AP-1, has been implicated in the inhibition of CD1d recycling and antigen presentation to NKT cells [67].

##### Betaherpesviruses

AP complex-related immunoevasive strategies have been well characterized for several members of the β herpesviruses family (Figure 2). For example, we noticed that MCMV protein m154 interferes with CD155 surface expression, in addition to the previously characterized MCMV m20.1 [129] and colocalizes with the AP-1-positive compartment [70]. m154 was already shown to reduce the cell surface expression of the SLAM family member CD48, suggesting the possibility of a broader mechanism of action of this viral protein [130] Indeed, we have identified that m154 targets large number of immunologically relevant cell surface molecules via interfering with their sorting at the level of AP-1 complex. Importantly, this modulation of AP-1 protein sorting impaired both NK and CD8 + T cell responses as an efficient immune-evasive strategy [70]. The same accumulation of CD155 in the AP-1 compartment was shown in HCMV infected cells, but the viral regulator was not determined.

Similarly, the HCMV glycoprotein UL20 interacts with AP-1 via a dileucine-based motif and is sorted into lysosomes leading to its rapid degradation [72]. Although the authors hypothesize that UL20 employs this strategy to sequester some cellular proteins to lysosomes for degradation, no specific cellular targets are identified so far.

The best studied example of β herpesvirus-AP interaction is the manipulation of MHC I. AP-1 and AP-3 complexes sort MCMV gp48/MHC I from the TGN to early endosomes and from early endosomes to lysosomes, respectively, preventing MHC I expression at the plasma membrane [46]. Similarly, the AP-2 complex mediates endocytosis of the m04/MHC I complex. The authors hypothesized that m04 acts as “adapter adapter”, connecting plasma membrane MHC I to AP-2 by simultaneous binding to both, which was supported by identification of YRRF motif in its cytoplasmic tail. Finally, enhanced endocytosis of the inhibitory ligand m04-MHC I was shown to prevent NK cell inhibition [71]. Regarding other members of the β herpesviruses family, it has been shown that both HHV-6 and -7 encode membrane glycoprotein U21, that reroutes newly synthesized MHC I molecules to the lysosomal compartment for degradation by utilizing both AP-1 and AP-3 [47]. Although the authors initially postulated that the mechanism of this regulation is dependent on the sorting signals in the cytoplasmic tail of U21, others have shown that U21 can affect the trafficking of MHC I molecules even without the cytoplasmic tail [48]. This was suggestive of an existence of another cellular protein that associates with the U21/MHC I, but the search for such a protein has been so far unsuccessful [47].

##### Gammaherpesviruses

Unlike other members of the family, γ herpesviruses have growth transforming activity and are associated with the pathogenesis of certain human cancers. They are ubiquitous and persistent in human population, suggesting efficient employment of immunoevasive strategies, some of which have been described to involve AP complexes (Figure 2).

For example, it has been shown that EBV protein BILF1 can abrogate T cell recognition by enhancing MHC I internalization and degradation. Interestingly, the same effect was not observed for the most closely related BILF1 homologue in KSHV, ORF74 [131]. A follow-up study showed an additional mechanism of MHC I regulation by diversion of newly synthesized MHC I molecules and identified the critical role of BILF1 C-terminal domain for this function. However, as BILF1 does not contain any typical tyrosine or dileucine based sorting signals, it was difficult to predict which AP complexes might be involved. Hence, although the authors did coimmunoprecipitate BILF1 and AP-1, siRNA knockdown experiments did not show a role for any of the major AP complexes suggesting that BILF1 might bind to a coadaptor protein, which bind cargo proteins not directly recognized by AP complexes [73].

Downregulation of MHC I via endocytosis has also been described for another member of γ herpesviruses, KSHV. Two viral proteins, MIR1 and MIR2 (encoded by ORFs K3 and K5), were shown to promote MHC I endocytosis and its degradation within endolysosomes [132]. In addition to MHC I, selective targeting of the costimulatory molecules B7.2 and ICAM-1 has been observed. Although it was initially suspected that the mechanism is similar to what is observed for HIV Nef interaction with AP complexes [133], later research showed that MIRs mediate ubiquitination of their targets followed by endocytosis and subsequent lysosomal degradation [134]. Finally, it has been suggested that KSHV also uses AP complex to promote viral entry. KSHV binds the receptor EphA2, and after series of signals including ubiquitination, AP-2 is recruited to promote viral entry via CME [74].

Similar to what has been observed for α herpesviruses, EBV encodes the BMRF-2 protein that directs viral particles to basolateral membranes and enhances cell to cell spread, potentially to protect the infectious virions from host immune surveillance. BMRF-2 is transported to the basolateral membrane by interaction with the AP-4 complex μ4 adaptin. Mutation of its tyrosine-based sorting motif YLLV, abolished its accumulation in the TGN, colocalization with AP-4 and transport to basolateral membranes, supporting its role in the interference with cell sorting machinery [57].

#### 2.2.3. Human JC Polyomavirus (JCV)

JCV belongs to the family of dsDNA polyomaviruses and causes a neurodegenerative disorder and progressive multifocal leukoencephalopathy by infecting oligodendrocytes and astrocytes and causing lytic destruction [135]. JCV enters the cells through CME, then uses Rab5 to get from early endosomes to caveolae-derived vesicles, showing a great example of hijacking the host endosomal trafficking network [136].

When it comes to the subversion of AP-mediated pathways, the only distinct interaction seemed to be through its agnoprotein [75]. JCV agnoprotein has been recognized as viroporin, a transmembrane class of viral proteins creating pores on host cell membranes and promoting virus replication [137]. JCV agnoprotein, which should be transported to lysosomal degradation by AP-3, essentially blocks this AP-3 function by binding its δ-adaptin. The agnoprotein mutant which fails to effectively bind δ-adaptin was transferred to lysosomes for degradation, implicating that, without the previously described interaction, agnoprotein is predetermined to be degraded before performing its function [75,137]. Interestingly, the agnoprotein is not the only viroporin shown to interact with adapter molecules but also the Vpu protein, viroporin encoded by HIV-1, interacts with the AP-1 complex to stop the antiviral molecule BST2/tetherin, from reaching the cell surface [100].

#### 2.2.4. Vaccinia Virus (VACV)

VACV, the most intensively studied member of the poxvirus family, is a large enveloped DNA virus that encodes for more than 200 proteins and replicates in the cytoplasm of the host cells [138]. VACV membrane fusion is accelerated by the acidic pH of the endosome, indicating that an endocytic pathway is used for virion entry into the cytoplasm [139,140]. Newly synthesized VACV envelope proteins are transported to the cell surface from where they are recycled and used by TGN or endosomal cisternae for optimal virion assembly [141]. In this regard, a dominant-negative form of Eps15, a component of clathrin-coated pits and vesicles associated with AP-2, increased the amounts of VACV envelope proteins in the plasma membrane and inhibited their internalization. Although the wrapping of virions appeared to be qualitatively unaffected, 50% reduction in released enveloped virions was observed, accompanied by a decreased formation of satellite plaques, and delayed virus spread. VACV envelope proteins have several motifs in their cytoplasmic domains known to recognize AP binding sites, with a documented interaction of VACV F13 protein with AP-2 [77,142]. 

The retrieval of viral proteins from the plasma membrane is an established salvage function for VACV virion assembly but may be also beneficial for reducing immune recognition of the infected cells.

#### 2.2.5. Coronaviridae

Severe acute respiratory syndrome coronavirus (SARS-CoV), Middle East respiratory syndrome-related virus (MERS) and SARS-CoV-2 are enveloped positive-sense RNA viruses characterized by club-like spikes that project from their surface and a large RNA genome. CoVs cause a variety of diseases in mammals and birds as well as lethal human respiratory infections [143].

The viral spike (S) protein [144] has been demonstrated to bind ACE2 receptor in vitro and in vivo but lacks an internalization domain. Therefore, it interacts with SARS-CoV 3a protein, which harbors the canonical YxxΦ AP-2 internalization motif. Indeed, the endocytosis of SARS-CoV-2 S protein was reduced in the presence of two drugs that block CME: dynasore and Pitstop 2 [145]. Specific inactivation of AP-2 by targeting μ2 or by AAK1 kinase inhibitor reduced cell entry and infectivity of SARS-CoV and SARS-CoV-2 pseudoviruses [78]. Another inhibitor, ACA, that interrupts AP-2/µ2-virus interaction exhibited potent antiviral efficacy against coronaviruses in vitro and in vivo [7]. The mouse hepatitis virus (MHV), a common model for studying coronavirus disease, also enters cells via CME and AP-2 mediated pathway [80,81]. 

#### 2.2.6. Flaviviridae

##### Dengue Virus (DENV)

DENV infection is one of the most prevalent mosquito-borne viral diseases in the tropical and subtropical territories. DENV is an enveloped positive sense RNA virus with variable clinical manifestation, including asymptomatic dengue fever and more severe life-threatening dengue hemorrhagic fever and dengue shock syndrome [82].

Simultaneous tracking of single DENV particles and various endocytic markers revealed that DENV enters cells via CME [146,147]. The adaptin role was observed when DENV particles failed to enter the cells expressing a dominant-negative mutant of Eps15 [82], and AP-1/μ1A silencing resulted in less DENV RNA and virion production [83]. Regarding the phosphorylation of AP complexes, it was shown that GAK, AAK1, and BIKE kinases are required in early and late stages of DENV life cycle [87,148,149] since their targeting with several pharmacological compounds led to the suppression of DENV infection in vitro and in vivo.

Taken together, these studies provide evidence for a prominent role of AP complexes, in the pathogenesis of one of the most unpleasant and recurring human pathogens and identify individual substances that are capable of disturbing the AP-DENV interactions.

##### Hepatitis C Virus (HCV)

HCV, a positive single-stranded RNA virus, encodes a single polyprotein, which is proteolytically cleaved into three structural proteins (core and the glycoproteins E1 and E2) and seven nonstructural (NS) proteins [85]. HCV enters its target cells in liver via CME following its binding to several cellular receptors (such as CD81, highly sulphated heparan sulphate, the low-density lipoprotein receptor, scavenger receptor class B type I and claudin-1 [150]).

Binding of HCV to µ2 of AP-2 is a mechanism by which HCV hijacks endocytic pathways [85,86]. The role of AP-2 phosphorylation kinases AAK1 and GAK in HCV life cycle was confirmed by treating the cells with their inhibitors [87,151]. It was also shown that HCV core binds AP-2 complex [56].

The AP-4 complex mediates HCV release and, together with AP-1/μ1A promotes cell-free spread, and with AP-1/μ1B supports cell-to-cell spread [56]. Further on, the σ1C adaptin isoform of AP-1 is a dependency factor for the efficient infection of hepatoma cells [84]. The HCV NS2 binds the µ4 adaptin of AP-4 with the greatest affinity, followed by AP-1/μ1A and AP-1/μ1B. This sorting of viral particles is reminiscent of the mechanisms used by herpesviruses, with HCV surpassing it in the variety of adaptins used.

#### 2.2.7. Human Rhinoviruses (HRVs)

Members of Picornaviridae family, HRVs, the major cause of the common cold, are characterized by more than 100 circulating types and are classified into two groups based on cell receptor specificity. Minor group HRVs bind LDLR super-family, that contain tyrosine sequence motifs, which direct them into clathrin-coated pits and AP-2 mediated endocytosis, involving the coadaptor proteins epsin-1 and CALM/AP180 [41]. The major HRVs group utilizes CAM-1 as its anchor point at the host plasma membrane, but their entry pathway has not been revealed in detail [41,152]. Based on the inhibition of infection by the dominant negative dynamin-2 mutant, it was proposed to also follow CME. However, ICAM-1 lacks a typical AP-2 binding motif and even functions as a viral receptor if its cytoplasmic tail is replaced with a GPI-anchor [153]. Therefore, the involvement of AP-2 in uptake of some HRV remains to be elucidated, while other HRV manipulation of AP complexes has not been described.

#### 2.2.8. Zaire Ebolavirus (Common Ebola Virus (EBOV))

Highly pathogenic Ebolavirus genus is a member of the Filoviridae family of single-stranded (-) RNA viruses that often form characteristic filamentous enveloped virions [154]. Species of Ebolavirus are causative agents of hemorrhagic fever with lethality rates up to 89% [155,156]. 

EBOV entry is mediated by the glycoprotein GP. Operating with infectious EBOV requires a BSL-4 facility, thus, pseudovirions expressing Ebola GP or EBOV virus-like particles (VLPs) have been developed [42,157]. This probably led to a large discrepancy in the results investigating the cell entry of the virus. Macropinocytosis has been identified as an entry route for EBOV-VLPs [158,159] while others argued that EBOV entry is dependent on more than ten clathrin-coated vesicle proteins, including AP-2 and the coadaptor proteins DAB2 and Eps 15 [42,43,157] and that silencing of kinases AAK1 and GAK resulted in the reduction of EBOV RNA [87]. It was also reported that the inhibition of AAK1 and GAK protects against morbidity and mortality in murine models of EBOV infection [87].

Given the fact that currently approved drugs are efficient against Zaire Ebolavirus, designing a treatment effective against a common target shared by all Ebolavirus species is a promising strategy for broadening current therapeutic options.

#### 2.2.9. Crimean–Congo Hemorrhagic Fever Virus (CCHFV)

The Crimean-Congo hemorrhagic fever virus, belonging to the Bunyaviridae family causes acute febrile illness, hemorrhage, multiple organ failure and shock with considerable mortality rate [88]. Primary transmission occurs via tick bite but human-to human transmissions have made this pathogen an important nosocomial health concern [160]. 

Two treatments that interfere with clathrin-coated vesicle formation, hypertonic sucrose, and chlorpromazine, decrease expression of CCHFV nucleocapsid protein [161], and Pitstop 2 CME inhibitor significantly decreased CCHFV infection [162]. Further on, the treatment of cells with siRNAs lowering the synthesis of AP-2 α adaptins resulted in a significant reduction in CCHFV infection rate [88]. Apart from mentioning AP-2 as a potential factor of virus uptake, no other findings have been reported.

#### 2.2.10. Influenza A Virus (IAV)

Influenza viruses are respiratory pathogens that represent a significant threat to public health, despite the large-scale implementation of vaccination programs [163]. IAV is the prototype member of the Orthomyxoviridae that are characterized by segmented negative strand RNA genome and unlike most RNA viruses, IAV replicates in the nucleus [164]. The function of all ten virus-encoded proteins has been well elucidated with hemagglutinin (HA) being responsible for mediating the binding of virus particles to sialic acid on host cells [90].

Approximately 60% of influenza viruses enter target cells through CME [165]. Thus, IAV exploits clathrin-dependent and -independent entry pathways, like micropinocytosis [166,167], and there might be a compensation for the blockage of a particular uptake mechanism by another. The internalization-competent HA mutants form higher order complexes, and this clustering depends on the strength of the internalization signal [89]. The clustering of HAs bearing strong internalization signals appears to be mediated via binding to AP-2. The use of multiple entry pathways and diverse coadaptors such as epsin 1 and FFAR2, which interacts with AP2 via β-arrestin, suggests that more specific signals than the initial HA-sialic acid contact are necessary to trigger internalization and promote IAV infection [90,168,169].

Given the key battle of IAV with hosts, in which it is crucial for the virus to mutate HA in order to avoid immune recognition but retain the ability to bind cells, understanding the mechanisms of IAV entry is of paramount importance. In addition to HA, in a recent study, substitutions made in the IAV nucleoprotein YxxØ motif affected viral fitness in vitro and in vitro [7].

#### 2.2.11. Paramyxoviridae

The Nipah virus (NiV) is a highly pathogenic paramyxovirus causing severe and often fatal respiratory and neurological disease with a 70% mortality rate [170]. The viral envelope of NiV includes two glycoproteins, NiV-F and NiV-G, which are key players in invasion of host cells (reviewed in [171,172,173]). The NiV-F contains a tyrosine-based motif and its interaction with μ1A is critical for its somatodendritic sorting in neurons [92] where it becomes a substrate for proteolytical enzymes cathepsin L and B, abundant in somatodendritic region. The proteolytically activated NiV-F is coupled to NiV-G and as such delivered to the axonal terminal domain [92]. The escorting of NiV-F to its optimal cleavage compartment by AP-1 is a direct example of AP functions in the polarized sorting of viral glycoproteins that further supports transneuronal viral propagation.

Hendra virus (HeV), another zoonotic virus from the same genus Henipavirus, has been identified after causing fatal infections in horses and getting transmitted to humans. Both HeV and NiV encode for matrix M protein, important for viral assembly and virus particle budding [174]. β3-adaptin hinge domain of AP-3 binds both NiV- and HeV- M protein while inhibiting this interaction decreases virus particle production, indicating that M protein highjacks cellular trafficking pathways [91]. Among the better-known representatives of viral proteins that use AP-3 to direct its cellular trafficking are HIV Gag proteins, with similar role to protein M [49,61] and VSV-G that utilizes AP-3 to traffic to the cell surface [50].

#### 2.2.12. Human Respiratory Syncytial Virus (RSV)

RSV, which belongs to the Pneumoviridae family, is an enveloped, negative sense, ssRNA virus. The lipid envelope contains three viral glycoproteins: the major attachment protein G, the fusion protein F, and a small hydrophobic protein SH. The matrix protein M is believed to form a layer on the inside of the viral envelope. Cell attachment of RSV is mediated by G and F, which bind to cellular glycosaminoglycans [175].

Previous studies on RSV entry suggested that RSV fuses its membrane directly with the membrane of target cells. On the other hand, a targeted siRNA screen revealed clathrin light chain, Eps-15, and AP-2 as important cellular factors in RSV infection [94]. In addition to contact with the host cell, AP complexes are also involved in the assembly of the RSV virus [95]. AP-3 complex binds RSV M via its μ3-adaptin and is further stabilized by an interaction with the δ-adaptin, while point mutated derivatives of RSV M in its conserved YxxL sorting motif impair the ability of RSV M to traffic to proper sites of viral assembly [95]. 

#### 2.2.13. Indiana Vesiculovirus (Former Vesicular Stomatitis Indiana Virus (VSIV or VSV))

VSV is a zoonotic, nonsegmented negative-strand RNA virus from the family of Rhabdoviridae and is shown to be less pathogenic than its cousin, the rabies virus [176]. VSV glycoprotein G interacts with δ-adaptin of AP-3 via YTDIE motif, but the VSV-G transport could not be completely abolished in the absence of AP-3 [50]. The reported findings on the interaction of VSV with AP-2 during cell entry are also somewhat contradictory [96,177].

#### 2.2.14. Retroviridae

##### Ecotropic Murine Leukemia Virus (eMuLV)

A member of retroviruses, particularly belonging to gammaretroviruses, eMuLV, can, as its name implies, cause cancer in mice after integration of its genome to the host DNA. As a retrovirus it has the usual Gag-Pol-Env structure [178] and the emergence of viruses associated with xenotropic mouse leukemia has shown that human cells can also be infected [179].

eMuLV reduces the surface expression of its receptor, the CAT-1, after infection-like other retroviruses that actively recruit receptors during cell entry. eMuLV infection decreased the association of CAT1 with AP-1, which was replaced by colocalization with AP-3. The only eMuLV protein found to interact with the CAT1 is its envelope (Env) protein and the expression of eMuLV Env alone was sufficient to increase the association of CAT1 with AP-3 [97]. The pattern is reminiscent of viruses that regroup target host molecules into lysosomes, for which the association with AP-3 is optimal.

##### Human Immunodeficiency Virus Type 1 (HIV-1)

HIV infection is characterized by a steady decline in the number and function of CD4+ T cells. The human-HIV interactome has revealed remarkable 500 interactions involving over 400 human proteins [180]. The main factor of virulence and the main element influencing membrane traffic is the Nef protein [98,99]. With such a thoroughly studied lifelong pathogen, one can expect numerous examples of manipulating the immune response, and AP complexes have once again proven to be an excellent choice.

**Figure 3:**
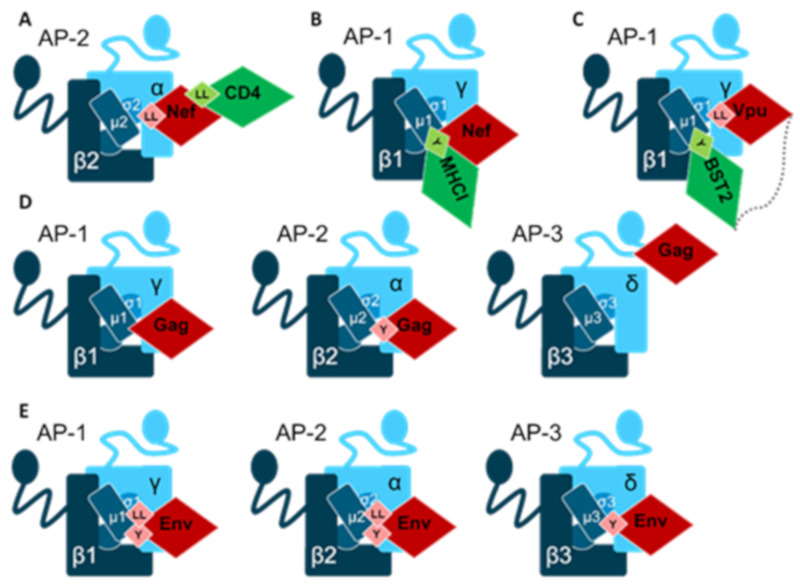
HIV-1 virus proteins (Nef, Vpu, Gag and Env) and their documented interactions with adaptins. Nef was shown to interact with AP-2 (**A**) and AP-1 (**B**), Vpu with AP-1 (**C**), while Gag (**D**) and Env (**E**) can interact with AP-1, 2 and 3. In case the motif participating in the interaction is known it is indicated (Y for tyrosine and LL for dileucine motif). Adaptins are shown in blue, HIV proteins in red, and molecules targeted by viral proteins are shown in green. Other molecules affected by HIV-1 are listed in the text.

Most research has been conducted on how Nef removes CD4 from the surfaces of infected cells, where Nef accelerates AP-2-mediated CD4 endocytosis (Figure 3A) [181,182,183]. CD4 has a dileucine signal whose binding to the AP-2 has been described; however, during infection, the interaction of the AP-2 and Nef proteins becomes dominant and the α-σ2 dimer:Nef interface is formed [184,185,186]. The crystal structure of the Nef and AP-2 was the final confirmation that Nef binds with highest affinity to σ2, then to α-adaptin, with Nef’s dileucine motif at the center of the binding [187]. This was followed by the crystal structure of Nef/AP2/CD4 which confirms how Nef functions as a connector between AP-2 and CD4 [185]. Given the current focus—on how to induce the death of latently-infected cells—it is particularly interesting that disruption of the Nef-AP-2 interaction may lead to CD4+ T cell apoptosis [188]. 

The second most interesting molecule that HIV Nef removes from the surface is the MHC I [189]. This is a logical choice for the virus because by reducing the amount of MHC I on the surface Nef reduces the ability of CD8+ T cells to recognize infected cells via MHC/viral peptides. This time, the mechanism is based on the AP-1 protein and the formation of the AP1-Nef-MHC I complex [190] although alternative pathways for Nef-mediated regulation of MHC-I are also described [99]. Binding of the Nef protein to the μ1A of AP-1 was demonstrated as early as 25 years ago, together with the fact that MHC I molecules of type HLA-A and -B are susceptible to Nef activity [191,192]. Nef binding to the MHC-I results in conformational change and represents a strong stabilizing factor for the binding of MHC I and AP-1 (Figure 3B) [190,193,194,195]. However, the same tyrosine motif, present in HLA A and HLA B molecules but not in HLA C, is important for cross-presentation of MHC I molecule via AP-1, thus confirming the functionality of the natural interaction between AP-1 and MHC I [196]. Once again, Nef took advantage of the natural route of sorting cellular proteins by solidifying existing complexes with APs and interfering with their proper expression on the cell surface.

Nef uses the described AP-2 subversion to downmodulate many other cellular proteins important in the antiviral immune response in addition to the CD4 molecule, which include the CD8, CD28, SERINC3, SERINC5 and, to some extent, CD3 molecules [197,198,199,200,201]. The binding of Nef to the AP-2 on the plasma membrane promotes its internalization and the degradation of its target proteins in lysosomes [98]. In the spectrum of proteins affected by Nef, AP-1 is responsible for fewer targets than AP-2, and it has been described that, in addition to regulating MHC I, Simian immunodeficiency virus (SIV) and some types of HIV affect tetherin via AP-1 [202]. AP-1 stable membrane binding depends on small GTPase Arf1, but the importance of Arf1:GTP in the regulation of AP-1 by Nef was not entirely clear [203,204,205]. It has finally been shown how Nef drives AP-1:Arf1 inner layer multimerization, which stimulates, accelerates, and stabilizes the formation of clathrin cages, and Nef achieves its goal–the clathrin-mediated subversion of targets and their induced lysosomal degradation [206].

BST2/tetherin is a molecule with a fascinating structure-an arc-like protein inserted at two ends into a lipid bilayer-that inhibits virus replication by binding virions to cell surfaces. It is a known antiviral factor that interferes not only with HIV but over 20 viral species [207,208]. In 2008, Vpu was found to colocalize with BST2/tetherin and inhibit its ability to retain virions on the surface of infected cells [209,210]. When the crystal structure of the ternary complex containing AP-1 and the cytoplasmic domains of BST2 and Vpu was resolved, Vpu was found to bind to the γ1- and σ1-adaptins of AP-1, thereby increasing the ability of μ1 to bind BST2 (Figure 3C) [100]. No direct binding of the cytoplasmic domains BST2 and Vpu has been established, but it is known how these proteins interact with the helix-helix interactions of their transmembrane domains [211]. Given that it has been shown how Vpu can interact with multiple subunits of both AP-1 and AP-2, it is possible to expect new targets and new mechanisms [100,212].

Gag protein is the major structural protein of HIV-1, essential for virion assembly and release. It greatly affects cellular trafficking pathways, largely due to its interaction with AP complexes [101]. HIV-1 Gag interacts with δ-adaptin of the AP-3 complex and the obstruction of this interaction prevents Gag from reaching the multivesicular body compartment and inhibits virus particle formation [49,213]. Although, an interaction has been proposed between the Gag matrix (MA) subunit and the hinge region of δ adaptin (Figure 3D), the subsequent production of these recombinant proteins has so far not yielded a complex [214]. Gag MA seems to be the site of interaction with μ1 adaptin as well [215]. Another Gag processing product interacts with AP complexes—the matrix-capsid junction is the site of interaction with the AP-2 complex, where binding to the μ2 adaptin involves a tyrosine motif [216]. Exploitation of AP complexes during the formation of infectious virions could be a part of the coordinated action of Gag and Env proteins. Env protein is able to manipulate AP-1, AP-2, and AP-3 complexes by binding to µ-adaptins via its tyrosine and dileucine motifs (Figure 3E) [217,218,219].

In conclusion, all key HIV-1 virus proteins have the potential to affect AP complexes, with effects on AP-1, AP-2, and AP-3 complexes being described.

##### Human Immunodeficiency Virus Type 2 (HIV-2)

HIV-2 is one of two major types of HIV viruses and is predominantly found in western Africa [220]. Unlike HIV-1, HIV-2 seems to be less pathogenic with its longer asymptomatic phase and relatively slower progression to AIDS even if their modes of transmission and intracellular replication pathways are similar [221]. 

HIV-2 and SIV Nefs can downregulate the T-cell receptor (TCR)-CD3 complex from the cell surface in contrast to HIV-1, even though the basic structure of all Nefs is similar [102,222]. In this case Nef employs AP-2 to mediate endocytosis of the TCR-CD3 complex.

HIV-2 Nef is not the only protein of this virus to interact and use AP-2 complex. The envelope (Env) protein GYxxθ motif uses AP-2 to enter endocytic pathways. The disruption of Env-AP-2 interaction negatively affects virus assembly and particle release [103]. Whereas HIV-1 Gag has been shown to interact with AP-1, -2, and -3 [49,215,216], HIV-2 Gag polyprotein depends on AP-3 and AP-5 for its transport through the cell [61]. 

HIV viruses are actually an example of how some very similar viruses-and even very similar proteins can develop different modes of AP protein manipulation and how one should be careful with implying an analogy.

#### 2.2.15. Hepatitis B Virus (HBV)

HBV, a member of the Hepadnaviridae family, is a small, enveloped virus containing a partially dsDNA genome (so called Dane particle) [223]. The entry of HBV into host cells is initiated by binding the viral particle to the receptor NTCP, which is a liver-specific transporter [224]. After HBV attachment, NTCP is thought to trigger HBV endocytosis using a yet unknown mechanism [106,225] and additional receptors may assist in virus binding [226,227,228].

Several studies supported the use of CME by HBV for cell entry [223,229]. The HBV preS1 has been shown to interact with clathrin and AP-2 [105] while CME inhibitors decreased HBV entry [104,229]. Consistent with this result was involvement of AP-2 and the Eps15 coadaptor protein in the infection [106]. AP complexes are not currently associated with any other aspect of HBV biology.

## 3. Concluding Remarks and Future Perspectives

**Figure 4:**
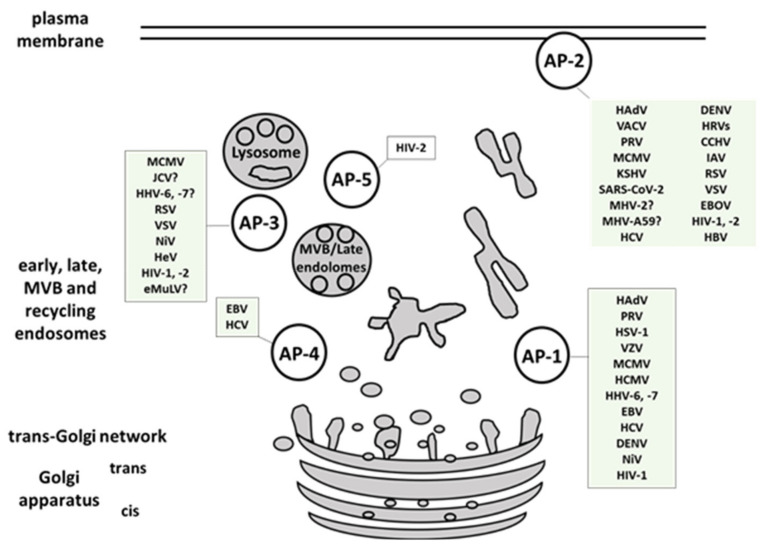
Multiple viruses exploit host AP complexes in different stages of their life cycles. AP-1 complex is involved in viral proteins intracellular trafficking, reduction of the surface MHC I molecules, release of viral particles and in viral replication. AP-2 complex is widely exploited for viral entry, and endocytosis of immune signaling molecules targeted by viruses. AP-3 is implicated in endolysosomal sorting and trafficking of viral proteins, and in viral particles production. AP-4 is shown to be important for viral replication, intracellular transport, and virion release. AP-5 mediates intracellular transport of HIV-2 Gag. MVB- multivesicular body. (For full virus species names refer to Table 1 or Abbreviations section.).

Viruses are obligate intracellular pathogens that rely on host cell machinery in various stages of their life cycles. A great number of host factors that are indispensable or contribute to successful viral infections has been identified. Multiple studies have shed light onto the unique strategies employed by individual viruses, but the accumulating data also indicate the overlapping patterns of viral-host interplay [86,230]. While different viruses necessitate distinct surface receptors to mediate successful attachment to the host cell, only a limited number of entry routes exists. It was established long ago that CME can be a part of the productive infectious cascade with the example of Semliki Forest virus [231]. Since then, numerous interactions of clathrin components and viral proteins or virions have been documented. During coevolution with host organisms, viruses have acquired genome sequences identical or closely resembling to motifs used by host proteins for the interaction with AP complexes. This enables them not only to maximize their own replication and assembly but importantly to interfere with the proper sorting and expression of host molecules hazardous to their survival. Here we presented a comprehensive overview of human viruses, including their most relevant animal models, that exploit members of the AP-family for their own trafficking, propagation, and avoidance of antiviral response (Table 1, Figure 4). AP-1 complex was found to be implicated in viral proteins intracellular trafficking, in reducing the surface expression of MHC I, in release of viral particles and even in viral replication. AP-2 complex is widely exploited for viral entry but also for endocytosis of immune signaling molecules targeted by viruses. AP-3 is involved in endolysosomal sorting and trafficking of viral proteins, as well as in viral particles production. AP-4 is shown to be important for viral replication, intracellular transport, and virion release, while AP-5 mediates intracellular transport of HIV-2 Gag. Among the discussed AP-interfering viruses, SARS-CoV and -2, CCHFV, EBOV, and NiV are listed in the WHO Research and Development Blueprint, an effort aimed at reducing the time of development and distribution of vaccines and treatments in public health emergencies. Identification and targeting of host trafficking pathways universal for unrelated viral pathogens is a promising approach to advance the discovery of new antiviral therapeutics. As an example, *N*-(*p*-amylcinnamoyl) anthranilic acid (ACA) was found to interrupt μ2-adaptin-virus interaction and exhibits in vivo activity against several viruses, including IAV, Zika virus, MERS, and SARS-CoV-2 [7]. Deeper understanding of virus-AP interplay is a premise for better characterization of currently known and yet uncovered host targets hijacked by multiple viral families. 

## Figures and Tables

**Table 1:** Viral families and their respective AP interaction complexes.

Group	Viral Family Members included in the Review			Viral Protein Involved	AP Complex Involved	References
dsDNA Viruses	Adenoviridae		Human Adenoviruses (HAdV, HAdV2, HAdV5)	RIDα, RIDβ	AP-1, AP-2	[62,63,64]
	Herpesviridae	α-herpesvirinae	Herpes simplex virus 1 and 2 (HSV-1, HSV-2)	gE/gI, VP22	AP-1	[65,66,67]
			Pseudorabiesvirus (PRV)	gB, gE	AP-1, AP-2	[68]
			Varicella zoster virus (VZV)	ORF9p	AP-1	[69]
		β-herpesvirinae	Murine Cytomegalovirus (MCMV)	m154, gp48, m04	AP-1, AP-2, AP-3	[46,70,71]
			Human Cytomegalovirus (HCMV)	UL20	AP-1	[72]
			Human herpes virus 6, -7 (HHV-6, -7)	U21	AP-1, AP-3	[47]
		γ-herpesvirinae	Epstein-Barr virus (EBV)	BILF1, BMRF,	AP-1, AP-4	[57,73]
			Kaposi’s sarcoma-associated herpesvirus (KSHV)	unknown	AP-2	[74]
	Polyomaviridae		Human polyomavirus (JCV)	agnoprotein	AP-2, AP-3	[75]
	Poxviridae		Vaccinia virus (VACV)	VACV F13, VACV A33	AP-2	[76,77]
ssDNA viruses (+ strand or “sense”)	not identified		/	/	/	/
dsRNA viruses	not identified		/	/	/	/
(+) ssRNA viruses (+ strand or sense)	Coronavirdae		Severe acute respiratory syndrome coronavirus (SARS-CoV, SARS-CoV-2)	unknown	AP-2	[7,78,79]
			Murine hepatitis virus (MHV)	unknown	AP-2	[80,81]
	Flaviviridae		Dengue virus (DENV)	unknown	AP-1, AP-2	[82,83]
			Hepatitis C virus (HCV)	NS2, NS5A, core	AP-1, AP-2, AP-4	[26,29,56,84,85,86]
	Rhinoviridae		Human rhinovirus (hRV)	LDLR (minor group)	AP-2	[41]
(−) ssRNA viruses (− strand or antisense) RNA	Filoviridae		Zaire ebolavirus (EBOV)	unknown	AP-1, AP-2	[43,87]
	Nairoviriade		Crimean-congo hemorragic fever (CCHFV)	unknown	AP-2	[88]
	Orthomyxoviridae		Influenza A (IAV)	Hemagglutinin	AP-2	[7,89,90]
	Paramyxoviridae		Hendra virus (HeV)	M-protein	AP-3	[91]
			Nipah virus (NiV)	NiV-F, M-protein	AP-1, AP-3	[91,92]
	Pneumoviridae		Human respiratory syncytial virus (RSV)	M-protein	AP-2, AP-3	[93,94,95]
	Rhabdoviridae		Vesicular stomatitis Indiana virus (VSV)	VSV-G	AP-2, AP-3	[50,96]
ssRNA-RT viruses (+ strand or sense) RNA with DNA intermediate in life-cycle	Retroviridae		Ecotropic murine leukemia virus (eMuLV)	Env	AP-3?	[97]
			Human immunodeficiency virus 1 (HIV-1)	Nef, Gag, Vpu	AP-1, AP-2, AP-3	[98,99,100,101]
			Human immunodeficiency virus 2 (HIV-2)	Nef, Env, Gag	AP-1, AP-2, AP-3, AP-5	[61,102,103]
dsDNA-RT viruses DNA with RNA intermediate in life-cycle	Hepadnaviridae		Hepatitis B virus (HVB)	preS1	AP-2	[104,105,106]

## Abbreviations

AMPA	α-Amino-3-hydroxy-5-methyl-4-isoxazolepropionic acid
ACA	*N*-(p-amylcinnamoyl) anthranilic acid
AAK-1	adaptor-associated protein kinase 1
AP	adaptor-protein-complex
ADP	adenosine 5′-diphosphate
Arf	ADP-ribosylation factor
ACE2	angiotensin converting enzyme 2
AIDS	acquired immunodeficiency syndrome
ATG9A	autophagy-related protein 9A
BSL	biosafety level
BST2	bone Marrow Stromal Cell Antigen 2
BIKE	bone morphogenic protein 2-inducible kinase
CAT-1	cationic amino acid transporter
CALM	clathrin-assembly lymphoid myeloid leukemia protein
CME	clathrin-mediated endocytosis
CCHFV	Crimean-Congo hemorrhagic fever virus
GAK	cyclin G-associated kinase
DENV	Dengue virus
EBOV	Ebola virus
eMuLV	Ecotropic murine leukemia virus
EGFR	epidermal growth factor receptor
Eps15	epidermal growth factor receptor substrate 15
EBV	Epstein-Barr virus
EXOC6	exocyst complex component 6
FFAR2	free fatty acid receptor 2
FDA	Food and Drug Administration
GPI	glycosylphosphatidylinositol
GAP	GTPase-activating proteins
GTP	guanosine-5’-triphosphate
HA	hemagglutinin
HeV	Hendra virus
HBV	hepatitis B virus
HCV	hepatitis C virus
HSV	herpes simplex virus
HAdV	human Adenoviruses
HHV	human betaherpesvirus
HCMV	human cytomegalovirus
Huh-7 cells	human hepatocellular carcinoma cell line
HIV	human immunodeficiency virus
HRVs	human rhinoviruses
VSV	Indiana vesiculovirus
IAV	Influenza A Virus
ICAM-1	intercellular adhesion molecule-1
JCV	JC polyomavirus
KSHV	Kaposi Sarcoma-associated Herpesvirus
LDLR	low-density lipoprotein receptor
LAMP1	lysosomal-associated membrane protein 1
MHC	major histocompatibility complex
MPR	mannose-6-phosphate receptors
mRNA	messenger ribonucleic acid
MERS	Middle East respiratory syndrome-related virus
MCMV	mouse cytomegalovirus
MHV	Mouse hepatitis virus
NKT cells	natural killer T cells
NiV	Nipah virus
PIP	phosphatidyl-inositol phosphates
PRV	pseudorabies virus
PHHs	primary human hepatocytes
PREPL	polyl endopeptidase-like
Rab5	Ras-related protein Rab-5A
RBD	receptor-binding domain
RIDα	receptor internalization and degradation
RSV	respiratory syncytial virus
SARS-CoV	Severe acute respiratory syndrome coronavirus
SLAM	signalling lymphocyte activation molecule
SIV	Simian immunodeficiency virus
siRNA	small interfering RNA
NTCP	sodium taurocholate co-transporting polypeptide
SPG	spastic paraplegia protein
TGN	trans-Golgi network
TNF	tumor necrosis factor
TRAIL-R	tumor necrosis factor-related apoptosis-inducing ligand receptor
VACV	Vaccinia virus
VZV	Varicella Zoster Virus
VLPs	virus-like particles
WHO	World Health Organization
